# Mindfulness-Based Stress Reduction for lung cancer patients and their partners: Results of a mixed methods pilot study

**DOI:** 10.1177/0269216315572720

**Published:** 2015-07

**Authors:** Desiree G. M. van den Hurk, Melanie P. J. Schellekens, Johan Molema, Anne E. M. Speckens, Miep A. van der Drift

**Affiliations:** Radboud University Medical Centre, Nijmegen, The Netherlands

**Keywords:** Mindfulness-Based Stress Reduction, lung cancer, partners, psychological distress, mindfulness

## Abstract

**Background:**

Lung cancer patients and partners show high rates of impaired quality of life and heightened distress levels. Mindfulness-Based Stress Reduction has proven to be effective in reducing psychological distress in cancer patients. However, studies barely included lung cancer patients.

**Aim:**

We examined whether Mindfulness-Based Stress Reduction might be a feasible and effective intervention for patients with lung cancer and partners.

**Design:**

Mindfulness-Based Stress Reduction is a training in which mindfulness practices are combined with psycho-education to help participants cope with distress. In this mixed methods pilot study, questionnaires on psychological distress and quality of life were administered before, directly after and 3 months after the Mindfulness-Based Stress Reduction training, in combination with semi-structured interviews.

**Setting/participants:**

Patients with lung cancer and partners were recruited at one tertiary care academic medical centre. A total of 19 lung cancer patients and 16 partners participated in the Mindfulness-Based Stress Reduction training.

**Results:**

Most patients were diagnosed with advanced stage lung cancer. Vast majority completed the training. Those receiving anti-cancer treatment did not miss more sessions than patients who were not currently treated. Patients and partners felt positive about participating in a peer group and with their partner. Among participants no significant changes were found in psychological distress. Caregiver burden in partners decreased significantly after following Mindfulness-Based Stress Reduction. The qualitative analysis showed that the training seemed to instigate a process of change in participants.

**Conclusion:**

The Mindfulness-Based Stress Reduction training seemed to be feasible for patients with lung cancer and their partners. A randomized controlled trial is needed to examine the effectiveness of Mindfulness-Based Stress Reduction in reducing psychological distress in lung cancer patients and partners.

**What is already known about the topic?**

- Lung cancer patients and their partners report high levels of psychological distress.
- The effectiveness of Mindfulness-Based Stress Reduction (MBSR) in reducing psychological distress in cancer patients has mainly been studied in females who were diagnosed with breast cancer in the curative stage of the disease.
- It is unknown whether MBSR would also be feasible and effective for lung cancer patients.

**What this paper adds?**

- This study demonstrates that MBSR is a feasible intervention for patients with lung cancer and their partners.
- MBSR seems to instigate a process of change in lung cancer patients and their partners, in which they become more aware of and gain more insight into their thoughts, feelings and bodily sensations.

**Implications for practice, theory or policy**

- MBSR might be an effective intervention for patients with lung cancer and their partners.
- A randomized controlled trial is needed to examine the effectiveness of MBSR in lung cancer patients and partners.
- Qualitative research might be of added value to tailor interventions to particular populations.

## Introduction

The global cancer statistics show that lung cancer is the second most common cancer worldwide with estimated new cases for males being 17.6% and for females being 9% each year.^1^ Lung cancer is the leading cause of death by cancer worldwide. At the time of diagnosis, lung cancer is often locally or systemically advanced and overall 5-year survival is only 17%.^2^

Receiving a diagnosis of lung cancer is a major cause of psychological distress, such as anxiety and depressive symptoms.^3,4^ Carlson et al.^5^ found heightened levels of distress in 58% of lung cancer patients, which in turn decreases the quality of life.^6^

Partners of patients with lung cancer also suffer from psychological distress.^7–9^ Partners have to cope with the uncertainty regarding the prognosis, dealing with the emotional reactions of the patient and managing the patient’s medical care.^10^ Around 40%–50% of partners of lung cancer patients report negative emotional effects of caregiving and high levels of distress.^11,12^

Although many studies reported on psychological distress and impaired quality of life in lung cancer patients and partners, not much is known about the effectiveness of possible psychosocial treatments.^13^ In the last decade, mindfulness-based approaches have been studied as a psychosocial intervention to reduce anxiety and depressive symptoms in patients with cancer. Mindfulness is defined as moment-to-moment present awareness with an attitude of non-judgement, acceptance and openness.^14^ The Mindfulness-Based Stress Reduction (MBSR) training is an 8-week group training in which participants practise mindfulness.

Despite the growing evidence of the positive effects of MBSR on the quality of life and well-being of cancer patients, mindfulness interventions have hardly been applied in patients with lung cancer.^15–17^ A meta-analysis concluded that MBSR leads to significant improvements in anxiety and depressive symptoms among cancer patients.^16^ The majority of participants were patients with breast cancer. Lung cancer patients are mostly older, male and in general have a poor prognosis. In previous studies, MBSR was mainly offered after physical treatments to help patients recover and handle daily life with their families and jobs. Also, very little is known about MBSR in partners.^18^ Because the median survival time of lung cancer patients is short, mindfulness might be particularly relevant in terms of acceptance and improving quality of life. This might apply to both patients and their partners. The aim of this study was to investigate the following questions: (1) Is MBSR a feasible intervention for patients with lung cancer and their partners? (2) Is MBSR effective in reducing psychological distress in lung cancer patients and their partners?

## Materials and methods

### Design and setting

To explore the feasibility and effectiveness of MBSR in lung cancer patients and partners, a mixed methods pilot study was conducted, following the Good Reporting of a Mixed Method Study (GRAMMS) guidelines.^19^ The study was conducted in a tertiary care academic medical centre from January 2010 to December 2011. The local medical ethics committee indicated that no formal approval was required as the study was an uncontrolled study of an intervention already used in clinical care of other cancer patients and the administration of questionnaires already used in routine outcome monitoring (registration number CMO2010/057). No written informed consent was obtained. Patients and partners were informed by their physician and nurse practitioner, received an information leaflet and could take as much time as needed to decide whether to participate.

### Participants

We included patients who were (1) diagnosed with cytological or histological proven non-small cell or small cell lung cancer and (2) had completed or were still receiving treatment. Patients with early stage lung cancer were classified as curative, whereas patients with (locally) advanced cancer and non-curative treatment were classified as palliative. Patients and partners were invited together but were also allowed to participate on their own. Patients and partners were excluded when they (1) were <18 years of age, (2) were not able to understand or use the Dutch language, (3) had already participated in a mindfulness-based intervention, (4) had current and regular treatment by a psychologist or psychiatrist or (5) had participated in another psychosocial programme.

### MBSR

The MBSR training was based on the original programme as developed by Kabat-Zinn,^14^ which consists of eight sessions of 2.5 h each, a silent day and daily home practice assignments of 45 min per day. Each participant received a CD-set to guide home practice and a workbook with information of each session. During MBSR, a variety of formal and informal exercises were practised. The patients were invited to do the exercises within the limits of their personal abilities. To make the intervention more suitable for patients with lung cancer and their partners, psycho-education about grief was added. The instructors of the MBSR training were health professionals and qualified mindfulness trainers who maintained a personal meditation practice.

### Assessments

Assessments took place at baseline, after MBSR training and 3 months later. Participants filled out the following questionnaires.

#### Psychological distress

The Hospital Anxiety and Depression Scale (HADS)^20,21^ consists of a seven-item anxiety (HADS-A) and seven-item depression (HADS-D) subscale.

#### Quality of life

The European Organisation for Research and Treatment of Cancer (EORTC) Core Quality of Life Questionnaire for Lung Cancer (QLQ-LC13)^22^ consists of 13 items targeting specific symptoms associated with lung cancer (coughing, haemoptysis, dyspnoea, pain) and side-effects from conventional chemo- and radiotherapy.

#### Psychological stress reaction

The Impact of Event Scale (IES)^23,24^ is a 15-item questionnaire measuring intrusive experiences and avoidance of thoughts and images associated with the event.

***Worry*** is measured with the 15-item Penn State Worry Questionnaire (PSWQ).^25,26^

***Lapses of Attention/Awareness*** are measured with the 15-item Mindful Attention and Awareness Scale (MAAS).^27^

#### Caregiver appraisal

The Self-Perceived Pressure from Informal Care (SPPIC)^28^ is a nine-item questionnaire, which assesses the extent to which caregiving is experienced as a burden.

***The Care-Derived Self-Esteem*** of the Caregiver Reaction Assessment (*CRA-SE*)^29^ was added to also assess positive aspects of caregiving.

**Figure 1.**
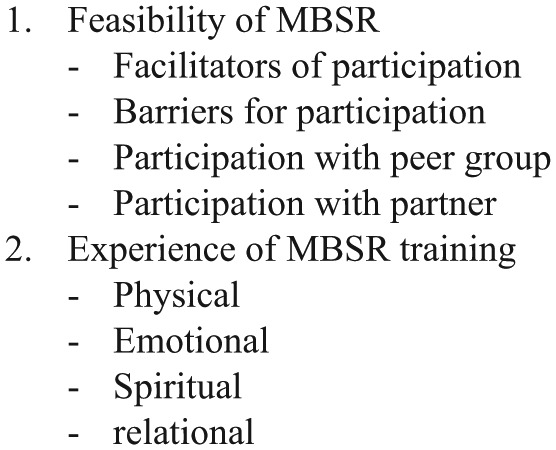
Topic list of semi-structured interview.

Within 1 year after completion of the MBSR training, semi-structured face-to-face interviews were conducted to explore participants’ experiences of the MBSR training. See Figure 1 for the topic list. Patients and partners were interviewed separately by a researcher who was not involved with the MBSR training. Interviews were audio-taped and transcribed in verbatim. These data were complemented by written evaluations at the end of the MBSR training.

By means of the questionnaires, the interviews and evaluation at the end of the MBSR training, triangulation was used to increase the reliability and validity of the results.

### Statistical and qualitative analysis

Paired *t*-tests were performed on an intention-to-treat basis to examine the difference between scores at baseline, directly after and 3 months after the MBSR programme. Interviews were analysed according to the thematic analysis approach.^30^ Three researchers read and coded the transcripts independently to minimize subjectivity. Next, codes were compared and discussed to assure consistency of coding. Together, the researchers grouped codes into themes.

## Results

### Study population

A total of 19 patients and 16 partners participated in the MBSR training. Clinical characteristics are summarized in Table 1. The majority of patients were diagnosed with advanced stage lung cancer (79%). Two and a half years after conducting the study, 13 patients (68%) had died.

**Table 1.** Clinical and psychological characteristics among patients and partners at baseline.

Clinical characteristics	Patients						Partners					
	Total (*n* = 19)		Completers (*n* = 16)		Non-completers (*n* = 3)		Total (*n* = 16)		Completers (*n* = 13)		Non-completers (*n* = 3)	
Age, mean (range)	61.7	(54–77)	62.2	(54–77)	59	(57–62)	60.9	(30–76)	59.8	(30–76)	65.0	(61–68)
Female gender, *n* (%)	9	(47)	7	(44)	2	(67)	9	(56)	9	(69)	0	(0)
Cancer type, *n* (%)												
Non-small cell lung cancer	15	(79)										
Small cell lung cancer	4	(21)										
Cancer stage, *n* (%), curative/palliative	4/15	(21/79)										
I	0	(0)	0	(0)	0	(0)						
IIa	2	(11)	2	(12)	0	(0)						
IIb	0	(0)	0	(0)	0	(0)						
IIIa	3	(16)	3	(19)	0	(0)						
IIIb	3	(16)	2	(12)	1	(33)						
IV	11	(58)	9	(56)	2	(67)						
Treatment(s) during MBSR, *n* (%)	14	(74)	11	(69)	3	(100)						
Chemotherapy	11	(58)	9	(56)	2	(67)						
Radiation	2	(11)	1	(6)	1	(33)						
Chemotherapy and radiation	1	(5)	1	(6)	0	(0)						
Time since diagnosis (in months), mean (range)	8.4	(2–35)	9.0	(2–35)	5.3	(3–9)						
Psychological distress (HADS), mean (SD)	14.7	(5.6)	14.6	(6.1)	15.7	(2.9)	17.7	(7.8)	18.5	(8.1)	14.1	(5.3)
Anxiety	7.8	(2.9)	7.9	(3.1)	7.7	(2.3)	9.9	(5.0)	10.3	(5.1)	8.0	(3.5)
Depression	6.8	(3.6)	6.7	(3.8)	8.0	(2.6)	7.8	(3.3)	8.2	(3.4)	6.1	(1.8)
Quality of life (QLQ-LC13), mean (SD)												
Dyspnoea	5.5	(2.0)	5.7	(2.1)	4.3	(1.5)						
Coughing	1.8	(0.9)	1.9	(0.9)	1.3	(0.6)						
Haemoptysis	1.2	(0.5)	1.2	(0.5)	1	(0.0)						
Pain	4.9	(1.5)	4.9	(1.3)	5	(2.6)						
Fatigue (CIS-F)	37.8	(12.0)	36.6	(12.0)	46.5	(0.7)						
Distress thermometer, mean (SD), *n* = 13	4.3	(1.97)										
Caregiver burden (SPPIC), mean (SD)							24.9	(6.0)	25.2	(5.6)	19	(5.7)

MBSR: Mindfulness-Based Stress Reduction; HADS: Hospital Anxiety and Depression Scale; SD: Standard Deviation; QLQ-LC13: Quality-of-Life Questionnaire–Lung Cancer 13; SPPIC: Self-Perceived Pressure from Informal Care; CIS-F: Checklist Individual Strength–Fatigue.

### Attendance of MBSR

A total of 16 patients (84%) and 13 partners (81%) attended four or more sessions of the MBSR training with a mean number of 7.9 (standard deviation (SD) = 1.5) out of nine sessions. Of patients who completed the training, 74% received chemo- and/or radiotherapy. There was no difference in mean number of sessions attended between patients receiving current anti-cancer treatment and those who were not treated during the training (7.4 (SD = 2.1) and 8.2 (SD = 1.3), n.s.). Of those who started the training, 13 patients (68%) and 11 partners (69%) partners completed the post-treatment assessment. The follow-up assessment was completed by 9 (47%) patients and 8 (50%) partners (Table 2).

**Table 2.** Baseline, post and follow-up scores of patients and partners.

	Patient						Partner					
	Baseline (*n* = 13; 68%)		Post (*n* = 13; 68%)		Follow-up (*n* = 9; 47%)		Baseline (*n* = 11; 69%)		Post (*n* = 11; 69%)		Follow-up (*n* = 8; 50%)	
	Mean	(SD)	Mean	(SD)	Mean	(SD)	Mean	(SD)	Mean	(SD)	Mean	(SD)
Psychological distress (HADS)	13.2	(5.9)	12.7	(5.7)	11.8	(7.7)	18.6	(9.1)	15.6	(7.0)	16.8	(8.8)
Anxiety	7.1	(2.4)	6.2	(2.7)	5.6	(3.8)	10.6	(6.8)	9.4	(4.0)	9.8	(4.0)
Depression	6.2	(4.0)	6.5	(3.9)	6.2	(4.3)	8.1	(3.9)	6.3	(3.6)	7.0	(5.0)
Quality of life (QLQ-LC13)												
Dyspnoea (items 3,4,5)	5.9	(1.9)	6.1	(2.1)	7.0	(2.8)						
Coughing (item 1)	2.0	(0.9)	2.1	(1.0)	2.3	(0.9)						
Haemoptysis (item 2)	1.2	(0.6)	1.2	(0.4)	1.0	(0.0)						
Pain (items 10, 11, 12)	5.0	(1.2)	4.7	(1.9)	4.4	(1.7)						
Fatigue (CIS-F)	35.9	(13.0)	33.6	(13.1)	34.9	(11.2)						
Psychological stress reaction (IES)												
Intrusive experiences	20.2	(7.8)	21.7	(5.1)	21.2	(4.1)						
Avoidance of thoughts	10.7	(4.1)	11.9	(3.4)	10.1	(2.8)						
(Worry) PSWQ	44.5	(15.0)	41.1	(12.3)	38.8	(6.8)	47.1	(10.0)	40.6	(17.2)	43.3	(8.3)
(Awareness) MAAS	67.5	(14.4)	65.1	(7.9)	67.8	(7.7)	64.0	(10.7)	59.6	(11.7)	60.9	(12.2)
Caregiver burden (SPPIC)							28.0	(3.6)	23.2	(3.8)*	21.1	(3.9)**
Caregiver self-esteem (CRA-SE)							30.2	(2.2)	30.0	(2.4)	30.6	(2.3)

SD: standard deviation; HADS: Hospital Anxiety and Depression Scale; QLQ-LC13: Quality of Life Questionnaire–Lung Cancer 13; CIS-F: Checklist Individual Strength–Fatigue; IES: Impact of Event Scale; PSWQ: Penn State Worry Questionnaire; MAAS: Mindful Attention and Awareness Scale; SPPIC: Self-Perceived Pressure from Informal Care; CRA-SE: Caregiver Reaction Assessment–Care-derived Self-Esteem.

* *p* < 0.05; **p < 0.01.

### Effectiveness of MBSR

No significant differences were found in pulmonary symptoms, fatigue and pain. Although mean scores of anxiety and depressive symptoms in both patients and partners decreased after MBSR, this change was not significant. Also, in both patients and partners, there were no significant changes in mindfulness skills and worry. The extent to which caregiving was experienced as burdensome by the partners decreased significantly after the MBSR training, both post-treatment and at follow-up.

### Qualitative evaluation

Of the patients who were alive and willing to participate in the qualitative evaluation, six patients (three males) and five partners (two males) were interviewed. A total of 66% of the patients had palliative stage lung cancer. Although we included all eligible patients who were willing to participate in the qualitative evaluation, saturation was not reached, because every interview still added new information.

### Facilitators and barriers

Patients mentioned that the duration and frequency of the training was feasible, despite their physical symptoms and current anti-cancer therapy. Most participants felt supported and facilitated by the mindfulness trainer. The folder and CDs were considered useful. Some people found it difficult to practise at home on a daily basis because of too much distraction. In addition to these general factors, three more specific subthemes emerged from the data, which could both function as facilitator and barrier. These themes included physical functioning, participation in a group and participation together with the partner. For corresponding quotations, see Table 3.

**Table 3.** Qualitative themes of facilitators and barriers and corresponding quotations of patients and partners.

	Examples of facilitators	Examples of barriers
Physical functioning	I liked the variety of exercises and could participate in all of them. (Patient)	There was a point in the exercise where I couldn’t keep up, and that was quite confronting, because a number of physical things, simple things, I couldn’t do anymore. (Patient)
Participating in a group	It gave me a liberating feeling to see that the others all had the same problem, you’re not alone, there are other people that have cancer. (Partner)	And then you start to think, ‘is it my turn now?’, that’s a big setback. (Patient)
Participating with partner	I heard how he dealt with it during the day, and that was nice. (Partner)	I couldn’t relax because I thought, he feels completely short of breath. (Partner)

#### Physical functioning

Physical functioning was mentioned by some patients and partners as a facilitator. One patient was surprised by his ability to participate in all the exercises. Other patients mentioned how physical limitations such as symptoms of fatigue or dyspnoea confronted them with being ill.

#### Participation in a group

Patients and partners felt positive about participating in a group, which felt as an open and safe environment. They felt connected with and supported by the group members. They also mentioned that they learned from others. Another person found it difficult to be confronted with the possible outcome of his disease by seeing other patients dropping out.

#### Participating with partner

Participation of both patients and partners in one group was perceived as helpful. They hoped they could support each other. They also encouraged each other to perform the exercises. Participating with a partner made it easier to talk to each other and with the children. It led to a better mutual understanding.

However, one partner and one patient felt worried and distracted during exercises about the well-being of their partner.

### Process of change

Although the process of change during MBSR was unique for every participant, we identified some aspects shared by most patients and partners. Based on the transcripts of the interviews, the following themes were identified: ‘standing still’, ‘being aware’, ‘insight’, ‘letting go’, ‘changing behaviour’ and ‘acceptance’. Participants moved back and forth between different aspects and not all participants experienced all of them. For the corresponding quotations, see Table 4.

**Table 4.** Qualitative themes of the process of change and corresponding quotations of patients and partners.

	Positive examples	Negative examples
1. Standing still	I got a more peaceful feeling more relaxed, a clearer mind. I always left with a good feeling. (Patient)	I couldn’t relax at all. (Partner)
2. Being aware	What do you do that you like and what do you do that you don’t like; how do you react to this and how could you react. Yeah, that’s what you do and what you notice. (Partner)	I don’t want to be continually reminded of it. A couple of weeks ago I thought, ‘I do have this disease, but I don’t feel anything’. I especially think that on good days. But then ten people say, ‘Yes, you are sick’. That is very difficult. (Patient)
3. Insight	Especially since I notice from myself that I tend to go on as if there’s nothing wrong. Just to feel as little as possible, because that makes it easier, no matter how difficult the situation is. (Partner)	
4. Letting go	That I can let go of more things, that I shouldn’t be occupied with it. That I think, ‘not now’. I used to be ‘go, go, go’ – I now have the peace so that I don’t have to rush. (Patient)	As long as you’re together, you’re together. I don’t leave him home alone. No, others might think completely different about it. A bystander says, ‘You should do other things’. I do that – I go shopping and go to friends. But to say now, ‘I’d like to go away for a few days’, no, then I wouldn’t be at ease. (Partner)
5. Changing behaviour	There came a time when it helped me to talk with my wife about it. It also got easier to talk with my children about it. […] That is a real joy. I can expose my feelings to my wife and vise versa. (Patient)	I can’t change myself anymore. It was also in the training: you have to do this, you have to do that … but it doesn’t work that way. My age probably plays a role – they say, ‘You have to do this’, but I don’t have to do anything. (Patient)
6. Acceptance	I can’t do much physically anymore. At the time, that was quite confronting, but that’s more than a half a year ago. There comes a time when you just have to accept it when it happens. (Patient)	If I’ve been awake for a half hour, then I know how my day will be. Will it be good or will I be extremely tired again. If I’m tired, then I just go from couch to couch (bench to bench). Then I get very angry at not being able to do anything. (Patient)

#### Standing still

The first component identified was ‘standing still’. By participating in the MBSR training, participants allowed themselves to stand still and to take time for themselves. For most participants, practising mindfulness led to inner rest and relaxation.

#### Being aware

Throughout the MBSR, patients noticed aspects they had not been aware of before. They described a greater awareness of their thoughts, emotions and physical sensations of the present moment. Participants were better able to allow their feelings and thoughts. However, some participants found it difficult to be confronted with their own negative emotions or thoughts, or those of their partners.

#### Insight in feelings

In a substantial number of participants, this increased awareness resulted in a greater insight in their thoughts, emotions and physical sensations. They started to recognize how they were related to one another and also how they tended to react to them. They began to become more aware of patterns in their behaviour.

#### Letting go

Some reactions started to change by letting go of thoughts and feelings rather than remaining stuck in them. By directing their attention to the present moment, they were better able to let go of worries about the future or sad ruminations about the past.

Some participants, however, were so caught up in their thoughts and feelings that it was difficult to let go. One patient blamed herself for having lung cancer and was angry with herself because of smoking cigarettes when she was younger. One partner was so focussed on the symptoms of her husband that she could not stop worrying about them.

#### Changing behaviour

With the newly gained insight into their habitual patterns, some participants were able to change their behaviour. Several started to make choices and set priorities which were more in line with their values. Patients and partners started to take better care of themselves. Another patient started to communicate more openly about the cancer with his wife and children.

A few participants could not change their behaviour. One patient explained how she could and would not change her behaviour, because she felt too old to change.

#### Acceptance

By letting go of their worrying thoughts and feelings, and changing their behaviour, some patients started to get to terms with the fact that they were ill and that their physical condition was worsening.

Other participants, however, were not able or did not want to accept their situation. They became frustrated every time they felt sick or ignored their symptoms.

In the evaluation of the training, patients and partners were positive about the increased awareness of the body, thoughts and feelings without judging. ‘Now I realize I am not the lung cancer disease itself, but the same woman now having a serious disease’. Some patients mentioned relief during a panic attack: ‘I discovered nothing serious happened and the feelings of anxiety diminished spontaneously’. Others mentioned more possibilities to become calm and take time for themselves.

## Discussion

This study showed that participating in MBSR was feasible for lung cancer patients and partners, despite the anti-cancer treatment and the advanced disease. The majority completed the MBSR, which is similar to a mean drop-out of 23% in former studies.^31^ Although no differences were found in psychological distress in patients and partners, the pressure of informal care experienced in partners decreased significantly after the MBSR. The qualitative analysis showed that the training seemed to instigate a process of change in patients and partners, in which they became more aware of and gained more insight into their thoughts, feelings and bodily sensations. This might be helpful in coping with this fatal disease.

A limitation of the study was the small sample size, which limits the power of the analyses. This means that the chance of a type II error occurring is heightened and the estimates of effect size are less reliable. Moreover, as a consequence of the small sample size, in the qualitative analysis, data saturation was not reached. Despite the small group of participants, the sample seemed to be representative of the global lung cancer population according to global cancer statistics, because the majority of patients were male and in the palliative stage of the disease.^1^

This study also had a number of strengths. Only a few studies have examined the feasibility and effectiveness of psychosocial interventions in lung cancer patients.^32^ Moreover, this is the first study also including partners of lung cancer patients in a psychosocial intervention study. This is quite surprising regarding the high rates of distress reported by both lung cancer patients and their partners.^4,7–10,12^ The few studies that did examine psychosocial interventions in lung cancer patients are promising. Temel et al.^6^ showed that early palliative care with a focus on psychosocial aspects can lead to improvement of quality of life and even life expectancy. Besides, the only study that examined the effectiveness of MBSR in both cancer patients and their partners showed that partners also benefit from participation.^18^

Additionally, we went beyond existing research by using both quantitative and qualitative methods to assess feasibility and effectiveness of MBSR in lung cancer patients and their partners.^19^ By adding the qualitative research to the quantitative data, insight was gained into the facilitators and barriers of participation, which helped us adapt the MBSR training for future use in a randomized controlled trial.^33^ For instance, additional attention is paid to physical limitations of patients during the gentle yoga exercises. Furthermore, at the start of the training, the trainer explicitly addresses the possibility of experiencing barriers (e.g. tendency of partners to worry about the patient and experiencing the stories of other participants as burdensome), which enables participants to become aware of these barriers and they are able to cope with them in an adaptive way. For the majority of the participants in our study, participating with the partner and a peer group functioned as facilitators of the training. These findings are in line with former research in breast cancer patients, describing the importance of peer support in MBSR.^34^ Therefore, we consider MBSR to be offered to both lung cancer patients and partners in a peer group setting.

This study shows the importance of studying lung cancer patients in psychosocial interventions. The majority of studies on mindfulness-based interventions have been conducted in female patients with breast cancer in the curative stage of the disease.^16^ Although lung cancer patients are difficult to study due to a poor prognosis and anti-cancer treatment, there is a need for tailored interventions.^32^ Moreover, our study also emphasizes the significance of including partners in psychosocial interventions for lung cancer. Not only because they often fulfil the role of family caregiver but also because they are exposed to the stressors of the lung cancer diagnosis, which can have a major impact on their physical and psychological well-being.

By showing that MBSR is a feasible intervention for lung cancer patients and partners, future research should set up a randomized controlled trial to examine the effectiveness of MBSR in lung cancer patients and their partners.^33^ Based on the process of change we identified, it may very well be that an MBSR training could not only reduce psychological distress but it can also support acknowledgement and acceptance of the disease and forthcoming death. It may even benefit the sharing between partners and the process of (anticipated) grief.

## References

[1] Jemal A Bray F Center MM Global cancer statistics. CA: Cancer J Clin 2011; 61: 69–90. 21296855 10.3322/caac.20107

[2] van der Drift MA Karim-Kos HE Siesling S Progress in standard of care therapy and modest survival benefits in the treatment of non-small cell lung cancer patients in the Netherlands in the last 20 years. J Thorac Oncol 2012; 7: 291–298. 22157366 10.1097/JTO.0b013e31823a01fb

[3] Stark DPH House A Anxiety in cancer patients. Br J Cancer 2000; 83: 1261–1267. 11044347 10.1054/bjoc.2000.1405 PMC2408796

[4] Brocken P Prins JB Dekhuijzen PNR The faster the better? A systematic review on distress in the diagnostic phase of suspected cancer, and the influence of rapid diagnostic pathways. Psychooncology 2012; 21: 1–10. 22905349 10.1002/pon.1929

[5] Carlson LE Angen M Cullum J High levels of untreated distress and fatigue in cancer patients. Br J Cancer 2004; 90: 2297–2304. 15162149 10.1038/sj.bjc.6601887 PMC2410292

[6] Temel JS Greer JA Muzikansky A Early palliative care for patients with metastatic non-small-cell lung cancer. N Engl J Med 2010; 363: 733–742. 20818875 10.1056/NEJMoa1000678

[7] Pinquart M Duberstein PR Optimism, pessimism, and depressive symptoms in spouses of lung cancer patients. Psychol Health 2005; 20: 565–578.

[8] Thielemann PA Conner NE Social support as a mediator of depression in caregivers of patients with end-stage disease. J Hosp Palliat Nurs 2009; 11: 82–90.

[9] Kim Y Duberstein PR Sorensen S Levels of depressive symptoms in spouses of people with lung cancer: effects of personality, social support, and caregiving burden. Psychosomatics 2005; 46: 123–130. 15774950 10.1176/appi.psy.46.2.123

[10] Mosher CE Jaynes HA Hanna N Distressed family caregivers of lung cancer patients: an examination of psychosocial and practical challenges. Support Care Cancer 2013; 21: 431–437. 22797839 10.1007/s00520-012-1532-6 PMC3493687

[11] Mosher CE Bakas T Champion VL Physical health, mental health, and life changes among family caregivers of patients with lung cancer. Oncol Nurs Forum 2013; 40: 53–61. 23269770 10.1188/13.ONF.53-61

[12] Ostlund U Wennman-Larsen A Persson C Mental health in significant others of patients dying from lung cancer. Psychooncology 2010; 19: 29–37. 19253315 10.1002/pon.1433

[13] Liao Y-C Liao W-Y Shun S-C Symptoms, psychological distress, and supportive care needs in lung cancer patients. Support Care Cancer 2011; 19: 1743–1751. 20949362 10.1007/s00520-010-1014-7

[14] Kabat-Zinn J Full catastrophe living: using the wisdom of your body and mind to face stress, pain and illness. New York: Delacorte, 1990.

[15] Foley E Baillie A Huxter M Mindfulness-based cognitive therapy for individuals whose lives have been affected by cancer: a randomized controlled trial. J Consult Clin Psychol 2010; 78: 72–79. 20099952 10.1037/a0017566

[16] Piet J Wurtzen H Zachariae R The effect of mindfulness-based therapy on symptoms of anxiety and depression in adult cancer patients and survivors: a systematic review and meta-analysis. J Consult Clin Psychol 2012; 80: 1007–1020. 22563637 10.1037/a0028329

[17] Carlson LE Doll R Stephen J Randomized controlled trial of mindfulness-based cancer recovery versus supportive expressive group therapy for distressed survivors of breast cancer (MINDSET). J Clin Oncol 2013; 31: 3119–3126. 23918953 10.1200/JCO.2012.47.5210

[18] Birnie K Garland SN Carlson LE Psychological benefits for cancer patients and their partners participating in mindfulness-based stress reduction (MBSR). Psychooncology 2010; 19: 1004–1009. 19918956 10.1002/pon.1651

[19] O’Cathain A Murphy E Nicholl J The quality of mixed methods studies in health services research. J Health Serv Res Policy 2008; 13(2): 92–98. 18416914 10.1258/jhsrp.2007.007074

[20] Zigmond AS Snaith RP The Hospital Anxiety and Depression Scale. Acta Psychiatr Scand 1983; 67: 361–370. 6880820 10.1111/j.1600-0447.1983.tb09716.x

[21] Spinhoven P Ormel J Sloekers PPA A validation study of the Hospital Anxiety and Depression Scale (HADS) in different groups of Dutch subjects. Psychol Med 1997; 27: 363–370. 9089829 10.1017/s0033291796004382

[22] Bergman B Aaronson NK Ahmedzai S The EORTC QLQ-LC13: a modular supplement to the EORTC core quality of life questionnaire (QLQ-C30) for use in lung cancer clinical trials. Eur J Cancer 1994; 30: 635–642. 8080679 10.1016/0959-8049(94)90535-5

[23] Horowitz M Wilner N Alvarez W Impact of Event Scale: measure of subjective stress. Psychosom Med 1979; 41: 209–218. 472086 10.1097/00006842-197905000-00004

[24] Brom D Kleber RJ De Schok Verwerkings Lijst [The impact of Event Scale]. Ned Tijdschr Psychol 1985; 40: 164–168.

[25] Meyer TJ Miller ML Metzger RL Development and validation of the Penn State Worry Questionnaire. Behav Res Ther 1990; 28: 487–495. 2076086 10.1016/0005-7967(90)90135-6

[26] van Rijsoort S Vervaeke G Emmelkamp P De Penn State Worry Questionnaire en de Worry Domains Questionnaire: eerste resultaten bij een normale Nederlandse populatie [The Penn State Worry Questionnaire and the Worry Domains Questionnaire: first results in a normal Dutch population]. Gedragstherapie 1997; 30: 121–128.

[27] Brown KW Ryan RM The benefits of being present: mindfulness and its role in psychological well-being. J Pers Soc Psychol 2003; 84: 822–848. 12703651 10.1037/0022-3514.84.4.822

[28] Pot AM Van Dyck R Deeg DJH Ervaren Druk door Informele Zorg: constructie van een schaal [Self-perceived pressure from informal care: construction of a scale]. Tijdschr Gerontol Geriatr 1995; 26: 214–219. 8750982

[29] Given CW Given B Stommel M The Caregiver Reaction Assessment (CRA) for caregivers to persons with chronic, physical and mental impairments. Res Nurs Health 1992; 15: 271–283. 1386680 10.1002/nur.4770150406

[30] Braun V Clarke V Using thematic analysis in psychology. Qual Res Psychol 2006; 3: 77–101.

[31] Ledesma D Kumano H Mindfulness-based stress reduction and cancer: a meta-analysis. Psychooncology 2009; 18: 571–579. 19023879 10.1002/pon.1400

[32] Schofield P Ugalde A Carey M Lung cancer: challenges and solutions for supportive care intervention research. Palliat Support Care 2008; 6: 281–287. 18662422 10.1017/S1478951508000424

[33] Schellekens MPJ Hurk DGM Prins JB Study protocol of a randomized controlled trial comparing mindfulness-based stress reduction with treatment as usual in reducing psychological distress in patients with lung cancer and their partners: the MILON study. BMC Cancer 2014; 14: Article 3. 10.1186/1471-2407-14-3 PMC3893473 24386906

[34] Mackenzie MJ Carlson LE Munoz M A qualitative study of self-perceived effects of mindfulness-based stress reduction (MBSR) in a psychosocial oncology setting. Stress Health 2007; 23: 59–69.

